# Rejuvenation of *Helicobacter pylori*–Associated Atrophic Gastritis Through Concerted Actions of Placenta-Derived Mesenchymal Stem Cells Prevented Gastric Cancer

**DOI:** 10.3389/fphar.2021.675443

**Published:** 2021-08-18

**Authors:** Jong Min Park, Young Min Han, Ki Baik Hahm

**Affiliations:** ^1^ College of Oriental Medicine, Daejeon University, Daejeon, Korea; ^2^ Western Seoul Center, Korea Basic Science Institute, Seoul, Korea; ^3^ Medpacto Research Institute, Medpacto, Seoul, Korea; ^4^ CHA Cancer Preventive Research Center, CHA Bio Complex, Seongnam, Korea

**Keywords:** *H pylori*, chronic atrophic gastritis, placenta-derived mesenchymal stem cell, rejuvenation, cell therapy, inflammation

## Abstract

Chronic *Helicobacter pylori* infection causes gastric cancer via the progression of precancerous chronic atrophic gastritis (CAG). Therefore, repairing gastric atrophy could be a useful strategy in preventing *H. pylori*–associated gastric carcinogenesis. Although eradication of the bacterial pathogen offers one solution to this association, this study was designed to evaluate an alternative approach using mesenchymal stem cells to treat CAG and prevent carcinogenesis. Here, we used human placenta-derived mesenchymal stem cells (PD-MSCs) and their conditioned medium (CM) to treat *H. pylori*–associated CAG in a mice/cell model to explore their therapeutic effects and elucidate their molecular mechanisms. We compared the changes in the fecal microbiomes in response to PD-MSC treatments, and chronic *H. pylori*–infected mice were given ten treatments with PD-MSCs before being sacrificed for end point assays at around 36 weeks of age. These animals presented with significant reductions in the mean body weights of the control group, which were eradicated following PD-MSC treatment (*p < 0.01*). Significant changes in various pathological parameters including inflammation, gastric atrophy, erosions/ulcers, and dysplastic changes were noted in the control group (*p < 0.01*), but these were all significantly reduced in the PD-MSC/CM-treated groups. Lgr5+, Ki-67, H^+^/K^+^-ATPase, and Musashi-1 expressions were all significantly increased in the treated animals, while inflammatory mediators, MMP, and apoptotic executors were significantly decreased in the PD-MSC group compared to the control group (*p < 0.001*). Our model showed that *H. pylori*–initiated, high-salt diet–promoted gastric atrophic gastritis resulted in significant changes in the fecal microbiome at the phylum/genus level and that PD-MSC/CM interventions facilitated a return to more normal microbial communities. In conclusion, administration of PD-MSCs or their conditioned medium may present a novel rejuvenating agent in preventing the progression of *H. pylori*–associated premalignant lesions.

## Introduction

There has been a broad paradigm shift in our understanding of gastric cancer prevention over the last three decades. Gastric cancers are the second leading cause of cancer-related deaths worldwide (Koulis et al., 2019), and their development is strongly associated with gastric ulcerations. The high number of gastric cancer deaths and the clear link between gastric cancers and *Helicobacter pylori* infections have led to the identification of several pathways for preventing pathogenesis. These include the possibility of reversing the pathogenesis of premalignant lesions by rejuvenating chronic atrophic gastritis (CAG), the total eradication of *Helicobacter* colonization and the concomitant reduction in mutagenic inflammation/oxidative stress, and the directed alteration of the tumor microenvironment and the mucosal immune response via engineering of the microbiota (Yashima et al., 2010; Chen et al., 2016; Liu et al., 2016) or some combination of these interventions. Most gastric carcinomas follow a documented and easily discernable cascade of precursor lesions, slowly progressing from the premalignant stages of CAG, to intestinal metaplasia (IM), and dysplasia to gastric carcinoma (de Vries et al., 2007; Moss, 2017), and since most of these lesions are a direct result of the chronic inflammation of gastric mucosa associated with *H. pylori* infection, multiple clinical interventions and trials have been implemented to prevent this cascade and detour the progression of this disease (Sipponen and Kimura, 1994; Correa and Piazuelo, 2012; Correa, 2013; Piazuelo and Correa, 2013; Rugge et al., 2013). These interventions all rely on the theory that gastric cancers associated with *H. pylori* infection can be prevented by the application of antioxidants or equivalent therapies via their reduction of the premalignant lesions including CAG with IM and their so-called suspension of the gastric precancerous cascade.

In addition to interventions such as antioxidants, phytoceuticals, and natural products, stem cells, including embryonic stem cells (ESCs), somatic cell–derived induced pluripotent stem cells (*i*PSCs), and mesenchymal stem cells (MSCs), are well-known therapeutic agents possessing unlimited self-renewal capacity and great potential to differentiate into various cell types from any of the three embryonic germ layers, including ectodermal, mesodermal, and endodermal lineages (Gumucio et al., 2008; Wang H. et al., 2020). Given this, we hypothesize that the administration of MSCs or their conditioned medium (CM) prior to irreversible dysplasia could facilitate the therapeutic rejuvenation of CAG. Therapeutic use of human placenta-derived MSCs (PD-MSCs) has been shown to exhibit enormous clinical potential as a source of regenerative medicine, with relatively low immunogenicity, easy producibility, and high stability. These attributes make MSCs uniquely qualified to support the regeneration of injured or diseased organs which is modeled by the CAG phenotypes in this study (Lu and Zhao, 2013; Quan and Wang, 2014).

The ability to replace defective cells in the stomach with cells that can engraft, integrate, and restore a functional epithelium could potentially cure atrophic gastritis. Stem cells or the factors included in their CM could serve as an attractive therapeutic strategy for dealing with *H. pylori*–associated gastric precancerous cascades. Efforts to identify efficient therapeutic agents or strategies capable of either rejuvenating *H. pylori*–associated gastric atrophy or preventing gastric cancer via their regulation of class I carcinogen *H. pylori* infection (Vogiatzi et al., 2007) remain a top priority. However, in addition to eradication, non-anti-microbial approaches, including the use of antioxidants, probiotics, vitamin E, *Artemisia*, and green tea, among others, have been evaluated. It is from these strategies that we selected the administration of PD-MSCs or their CM since multiple clinical trials involving MSCs in a range of human diseases or as the primary cell source in cell therapies and regenerative medicine strategies are already underway (Bunpetch et al., 2017; Kimbrel and Lanza, 2020; Maqsood et al., 2020; Shariati et al., 2020). In this study, we investigated whether PD-MSCs or their concentrated CM administered during *H. pylori*–associated CAG could induce a rejuvenating effect on CAG and facilitate a reversion of these lesions to their premalignant state while allowing us to explore the molecular mechanisms underlying these actions.

## Materials and Methods

### Cell Culture

PD-MSCs were obtained from CHA University (Prof. Yong Soo Choi, CHA University, Seongnam, Korea). The PD-MSC line was cultured in α-MEM containing 1 μg/ml heparin, 25 mg/ml fibroblast growth factor, 10% (*v*/*v*) fetal bovine serum, and 100 U/ml penicillin. The cells were maintained at 37°C in a humidified atmosphere containing 5% CO_2_. The rat gastric mucosal cells, RGM-1, were kindly given by Prof. Hirofumi Matsui (University of Tsukuba, Japan) and were maintained at 37°C in a humidified atmosphere containing 5% CO_2_. RGM-1 cells were cultured in Dulbecco’s modified Eagle’s medium containing 10% (v/v) fetal bovine serum, 100 U/ml penicillin, and 100 μg/ml streptomycin. For co-culture experiments, PD-MSCs were seeded in a six-well transwell system and cultured for 24 h before co-culture with RGM-1 cells. Just prior to co-culture, PD-MSCs were washed with PBS three times and co-cultured with RGM-1 in the RGM-1 cell culture medium.

### *H. pylori* Culture

**FIGURE 1:**
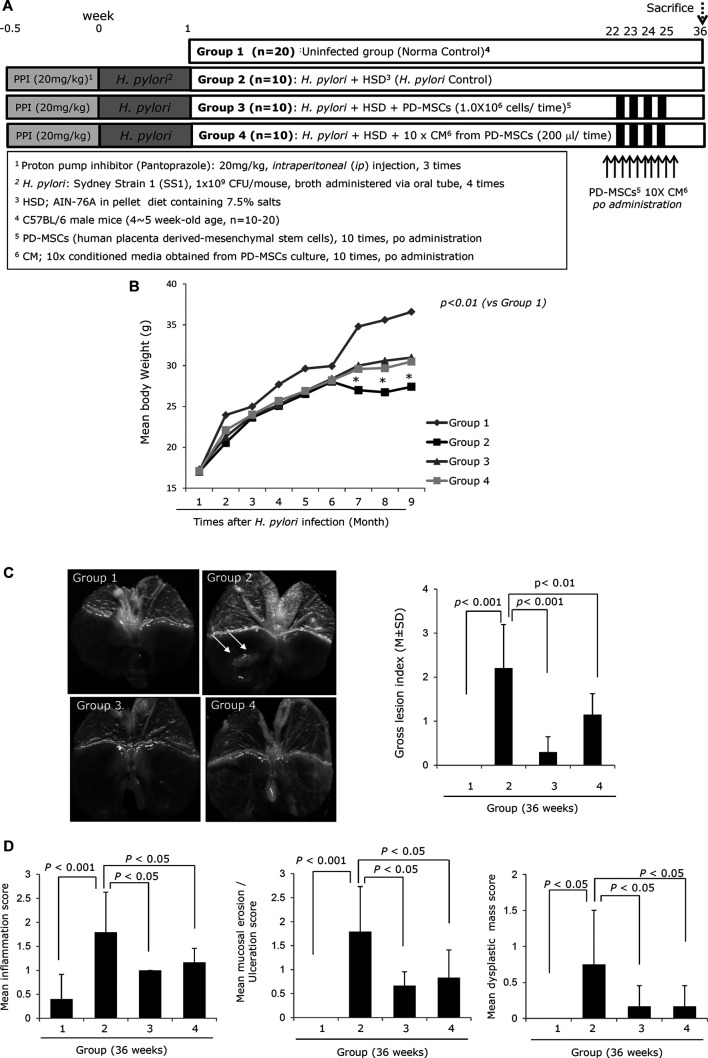
Influence of PD-MSCs or their CM on an *H. pylori*–initiated, high-salt diet–promoted CAG mice model (36 weeks). **(A)** Scheme for groups: Group 1, normal control; Group 2, *H. pylori*–associated CAG disease control; Group 3, disease control treated with 1x10^6^ PD-MSCs (100 μl), 10 times during 22–25 weeks; and Group 4, disease control treated with 10x concentrated CM (200 μl). **(B)** Body weight changes according to groups. Body weights were measured every 3 days in all mice. **(C)** Representative photo of the resected stomach and mean gross lesion scores according to groups, see Supplementary Table 1 for the scoring system. **(D)** Representational pathology and mean pathological scores according to groups, see Supplementary Table 2 for the scoring system. All data represent mean ± SD (*n* = 10).

*H. pylori* strain ATCC43504 (American Type Culture Collection, *cag*A+ and *vac*A s1-m1 type strain) was used for the *in vitro* cell model and Sydney strain (SS1, a *cag*A+ and *vac*A s2-m2 strain adapted for mice infection) for the *in vivo* model. *H. pylori* bacteria (Figure 1A and Figure 4A) were cultured at 37°C in a BBL Trypticase soy (TS) agar plate with 5% sheep blood (TSAII; BD Biosciences, Franklin Lakes, NJ) under microaerophilic conditions (BD GasPaK EZ Gas Generating Systems, BD Biosciences) for 3 days. The bacteria were harvested in clean TS broth, centrifuged at 3,000×*g* for 5 min, and resuspended in the broth at a final concentration of 10^9^ colony-forming units (CFUs)/ml. In all experiments, cultures grown for 72 h on TS agar plates were used.

### Animals and Study Protocol: Experimental Protocol of *H. pylori*–Infected Mice Model

Five-week-old male C57BL/6 mice (WT mice) were purchased from Orient (Seoul, Korea), and they were housed in a cage maintained in a 12 h/12 h light/dark cycle under specific-pathogen-free conditions (*n* = 50). C57BL/6 mice were purchase from Central Lab Animal Inc. (Seoul, Korea). Six-week-old female C57BL/6 mice were fed sterilized commercial pellet diets (Biogenomics, Seoul, South Korea) and sterile water *ad libitum* and housed in an air-conditioned biohazard room at a temperature of 24°C. We divided 50 mice into four groups: Group 1 (*n* = 10), WT mice in the vehicle control group; Group 2 (*n* = 20), WT mice in the *H. pylori*–infected disease control group; Group 3 (*n* = 10), WT mice in the *H. pylori*–infected disease group administered 1x10^7^/100 ml PD-MSCs; and Group 4 (*n* = 10), WT mice in the *H. pylori*–infected disease group administered CM obtained from PD-MSCs, 100 μl concentrated from PD-MSC culture. We maintained these four groups up to 36 weeks, respectively. All groups were given intraperitoneal injections of pantoprazole, 20 mg/kg (Amore-Pacific Pharma, Seoul, Korea), as the proton pump inhibitor (PPI), three times per week, to increase successful *H. pylori* colonization through lowered gastric acidity, and then, each mouse was intragastrically inoculated with a suspension of *H. pylori* containing 10^8^ CFUs/ml or with an equal volume (100 μl) of clean TS broth using gastric intubation needles. The *H. pylori*–infected mice were fed a special pellet diet based on AIN-46A containing 7.5% NaCl (high-salt diet, Biogenomics, Seongnam, Korea) for a total of 36 weeks (Figure 1A and Figure 4A) to promote the *H. pylori*–induced carcinogenic process in all infected animals. Randomized groups of mice (*n* = 10) were sacrificed after 36 weeks of *H. pylori* infection, respectively, based on our previous experience that CAG was generated at 24 weeks and gastric tumorigenesis was generated after 36 weeks (Park et al., 2014). The body weight was checked in all mice every 3 days up to observational periods. The stomachs of mice were opened along the greater curvature and washed with ice-cold PBS. The numbers of either erosions/ulcers or protruded nodule/mass were determined under the magnified photographs (Figure 1C). Stomachs were isolated and subjected to histologic examination, ELISA, western blotting, and RT-PCR. All animal studies were carried out in accordance with protocols approved by the Institutional Animal Care and Use Committee (IACUC) of CHA University, CHA Cancer Institute, after IRB approval (IRB 17-0901).

### Statistical Analysis

The results are expressed as mean (standard deviation (SD)). Statistical analyses were conducted with GraphPad Prism (GraphPad Software, La Jolla, CA) and SPSS software (version 12.0; SPSS Inc., Chicago, IL). Statistical significance between groups was determined by a multi-variate Kruskal–Wallis test. Statistical significance was accepted at *p < 0.05*.

### Supplementary Information

Detailed experimental procedures for gross lesion index, index of histopathologic injury, immunohistochemical staining, terminal deoxynucleotidyl transferase–mediated dUTP nick-end labeling (TUNEL) staining, RT-PCR, western blotting, cytokine protein array, preparation of cytosolic and nuclear extracts, RNA interference, zymography, bacterial DNA extraction from mouse stool samples, bacterial metagenomic analysis using DNA from stool samples, and analysis of bacterial composition in the microbiota can be found in the Supplementary Materials.

## Results

### Placenta-Derived Mesenchymal Stem Cells (PD-MSCs) or Their Conditioned Medium Ameliorates *H. pylori*–Associated CAG

**FIGURE 2:**
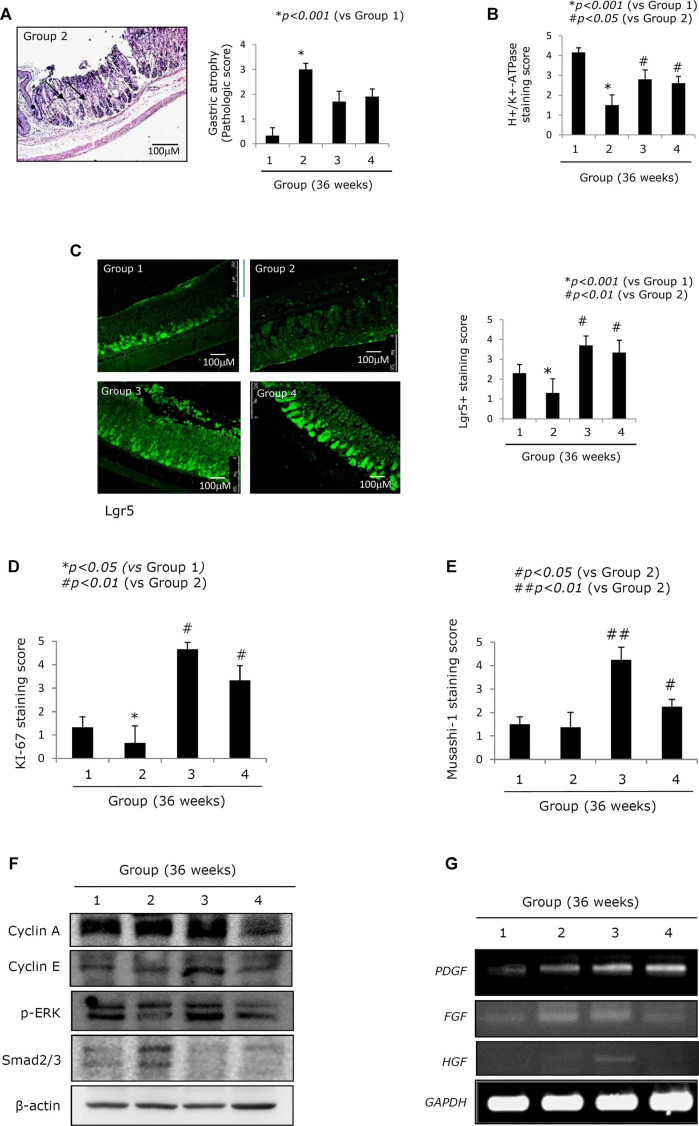
Changes of stemness according to groups relevant to gastric atrophy. **(A)** Scores for gastric atrophy: left, representational pathology of Group 2 showing significant changes of CAG featured with a loss of parietal cells, gastric inflammation, and a loss of gastric glands with some foci of erosions; right, scores according to groups. See Supplementary Table 2 for the scoring system. **(B)** Immunohistochemical staining of the proton pump with antibody of H^+^/K^+^-ATPase. **(C)** Confocal staining with LGR5+ antibody: left, representational staining with LGR5+, x100 magnification; right, mean scoring according to groups. **(D)** Immunohistochemical staining of Ki-67 antibody and the mean positive scoring according to groups. **(E)** Immunohistochemical staining of Musashi-1 antibody and the mean positive scoring according to groups. **(F)** Western blot for cell cycle, ERK among MAPK, and smad2/3. **(G)** RT-PCR for *PDGF*, *FGF*, and *HGF* mRNA. All data represent mean ± SD (*n* = 10).

Since we already have an established *H. pylori*–induced CAG mouse, which relies on an *H. pylori* infection–initiated, high-salt diet–promoted mouse model (Nam et al., 2004a; Nam et al., 2004b; Park et al., 2014; Jeong et al., 2015; Han et al., 2016; An et al., 2019), we went on to design this experiment to document the ameliorating action of PD-MSCs or their conditioned medium (CM) against *H. pylori*–induced CAG. We administered ten doses of PD-MSCs or their CM following 22 weeks of *H. pylori* infection and then evaluated these animals over a nine-week period until they were terminated for end point analysis at 36 weeks (Figure 1A). Our CAG model was initiated following four PPI injections, which facilitates the successful colonization of *H. pylori* in the lowered gastric pH and of cultured *H. pylori* into mice who were then fed a 7.5% salt AIN-76A pellet diet until 20–24 weeks, when the control mice (Group 2) showed significant changes in CAG, manifested with erosions, ulcers, and a very thin atrophied gastric wall (Figure 1C). To encourage the rejuvenating effects of the PD-MSCs (1 × 10^7^/100 μl PD-MSCs, Group 3) or their concentrated CM (100 μl, concentrated from PD-MSC culture), they were were administered to groups of mice via the *oral* route approximately 10 times before the mice were sacrificed at 36 weeks and subjected to histological examination. In addition, each mouse was evaluated for body weight over the total course of treatment as this is an effective marker for atrophic gastritis. Figure 1B clearly shows that the mean body weights of the *H. pylori* control mice significantly decreased between week 24 and the end point of the experiment (*p < 0.01*), while there were no significant changes in body weight in the groups treated with PD-MSCs or their CM. There were also significant changes in the gross lesions of the stomach in the control animals at 36 weeks, with these animals presenting with multiple scattered nodular and elevated mass-like lesions, thinned corpus and pylorus walls, and some scattered nodular changes, signifying the development of typical CAG. The gross lesion index revealed that there were significant changes in the *H. pylori*–infected stomach and that intervention with PD-MSCs or their concentrated CM (*p < 0.01*) significantly ameliorated the severity of these lesions (Supplementary Table 1; Group 4, *p < 0.01*, Figure 1C). The resected stomachs from each group were also subjected to pathological evaluation, and the total pathological scores describing gastric inflammation, gastric atrophy, and tumorigenesis were significantly decreased in the PD-MSC–treated group compared to the control group (Supplementary Table 2; *p < 0.001*, Figure 1D). Gastric atrophy is generally associated with a loss of parietal cells and gastric glands and increased inflammatory cell infiltration. Our evaluations revealed that Group 2 demonstrated a typical increase in these parameters (*p < 0.001*), but that intervention with PD-MSCs or their CM significantly ameliorated these effects (*p < 0.05*, Figure 2A; Supplementary Figure 1A). Further investigation of the proton pump in the parietal cells, analyzed via immunostaining of H^+^/K^+^-ATPase, revealed a significant decrease in its expression in the control group (*p < 0.01*) but an increase in its expression in Groups 3 and 4 (*p < 0.05*, Figure 2B; Supplementary Figure 1B). These results suggest that PD-MSCs and their CM exert a significant mitigating effect on *H. pylori*–induced CAG.

### Mitigated CAG in Response to PD-MSC Treatment Is Closely Associated With an Enrichment in the Number of Lgr5+ Cells

Lgr5+ cells have been identified as a possible source of stemness in the stomach (Hata et al., 2018; Sigal et al., 2019; Tang et al., 2019). Figure 2C shows that the expression of Lgr5+ was significantly decreased in Group 2 (*p < 0.05*, Figure 2C) and that there was a loss of leucine-rich repeat-containing G-protein–coupled receptor 5+ (Lgr5+) cells following chronic *H. pylori* infection. On the contrary, this trend was reversed following treatment with either PD-MSCs or their CM (*p < 0.01*, Figure 2C). Ki-67–mediated evaluation of cellular proliferation revealed that there were significantly fewer Ki-67 cells in the control group when compared to the healthy control. However, Groups 3 and 4 showed significant increases in Ki-67 expression (*p < 0.01*, Figure 2D; Supplementary Figure 1C). Musashi-1 expression in the stomach reflects stemness. Musashi-1 expression was increased in Groups 3 and 4 (*p < 0.05*, Figure 2E). In addition, the expressions of cyclin A, cyclin E, p-ERK, and smad2/3, which are all implicated in the pathogenesis of *H. pylori*–associated CAG, were evaluated in response to treatment with PD-MSCs or their CM. PD-MSCs significantly increased cyclin E expression, and PD-MSCs and their concentrated CM significantly decreased cell growth suppressive *smad*2/3 expression (Figure 2F; Supplementary Figure 2). The expression of *PDGF* mRNA in the resected stomach tissues was significantly increased in response to treatment with either PD-MSCs or their concentrated CM. The expression of HGF mRNA was increased in response to treatment with PD-MSCs (Figure 2G).

### PD-MSC Treatment Induces Preemptive Amelioration of Apoptosis and Protease Inhibition Alleviating CAG in the Mouse Model

**FIGURE 3:**
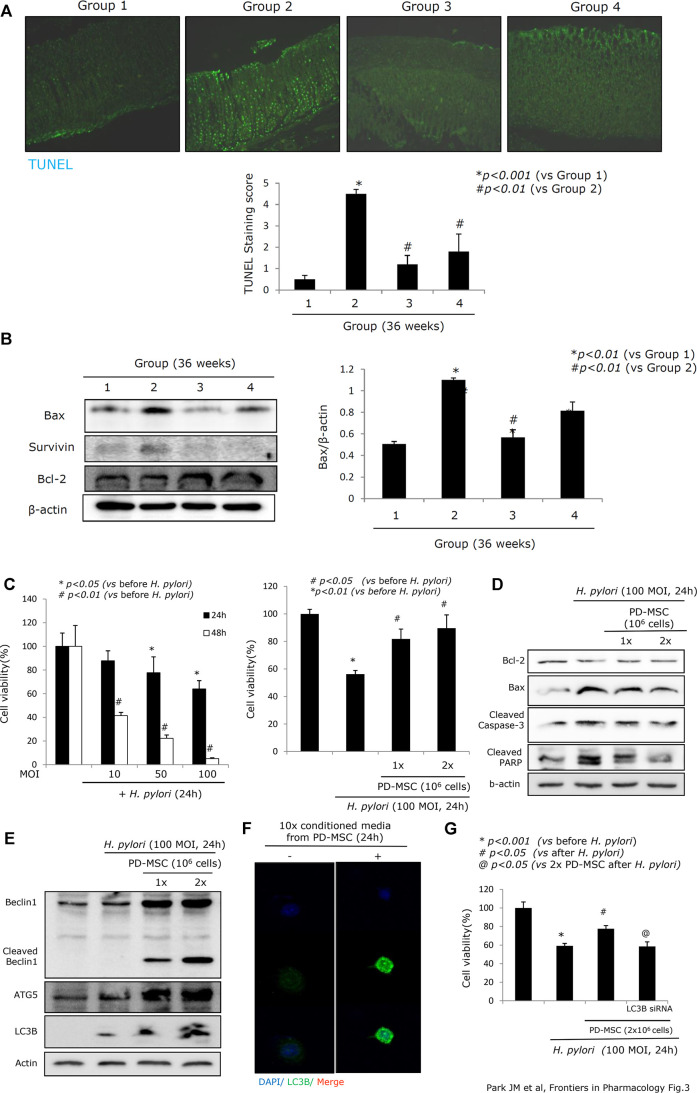
Apoptotic status according to groups. **(A)** TUNEL staining with apoptotic index according to groups, x100 magnification. **(B)** Western blot for apoptotic executors, Bax, surviving, and Bcl-2. **(C)** Changes of RGM-1 cells’ viability after *H. pylori* infection in a different time point and different POI in a transwell co-culture system. Cell counting using a hemocytometer and trypan blue for measuring cell viability was done after 24 hr of *H. pylori* infection in the absence or presence of PD-MSCs. **(D)** Western blot for apoptotic executors in the presence or absence of PD-MSCs under *H. pylori* infection, 100 MOI, 24 hr. **(E)** Western blot for autophagy, Beclin1, cleaved Beclin1, ATG5, and LC3B in the presence or absence of PD-MSCs under *H. pylori* infection, 100 MOI, 24 hr. **(F)** Confocal imaging of LC3B after CM administration. **(G)** Cell viability after PD-MSCs in the presence of *H. pylori* infection in mock cells and LC3B siRNA transfection. All data represent mean ± SD (*n* = 10).

Chronic *H. pylori* infection induces considerable levels of apoptosis, a loss of parietal cells in the corpus, decreased somatostatin-secreting D cells in the antrum, and robust apoptosis in the epithelial cells, which are responsible for atrophic gastritis, peptic ulcer disease, and mucosal erosions (Alzahrani et al., 2014; Zhao et al., 2020). Figure 3A shows that 36 weeks of chronic *H. pylori* infection led to considerable levels of apoptosis (*p < 0.001*). However, animals treated with PD-MSCs showed significantly decreased levels of apoptosis, even during chronic *H. pylori* infection (*p < 0.01*, Figure 3A). Western blot against the central apoptosis-related molecules showed a significant increase in Bax in response to CAG conditions (Group 2). However, the levels of Bcl-2 were significantly increased and Bax was significantly decreased in Group 3 (*p < 0.05*, Figure 3B). Given this, we extended our investigation to include an exploration of the anti-apoptotic effects of PD-MSCs using a transwell co-culture system. Figures 3C,D demonstrate that *H. pylori* infection significantly decreased RGM-1 cell viability, while the addition of PD-MSCs significantly decreased the expression of *H. pylori*–induced apoptotic executors. Further evaluations of the autophagy response in cells treated with PD-MSCs or their CM demonstrated that there was a significant increase in LC3B-II and ATG5 autophagosomes (Figures 3E,F; Supplementary Figure 3) and that this increase was lost in response to LC3B siRNAs (Figure 3G).

### PD-MSC–Mediated Preservation of 15-PGDH Afforded These Treatments’ Anti-Tumorigenic Effects and Alleviated CAG

**FIGURE 4:**
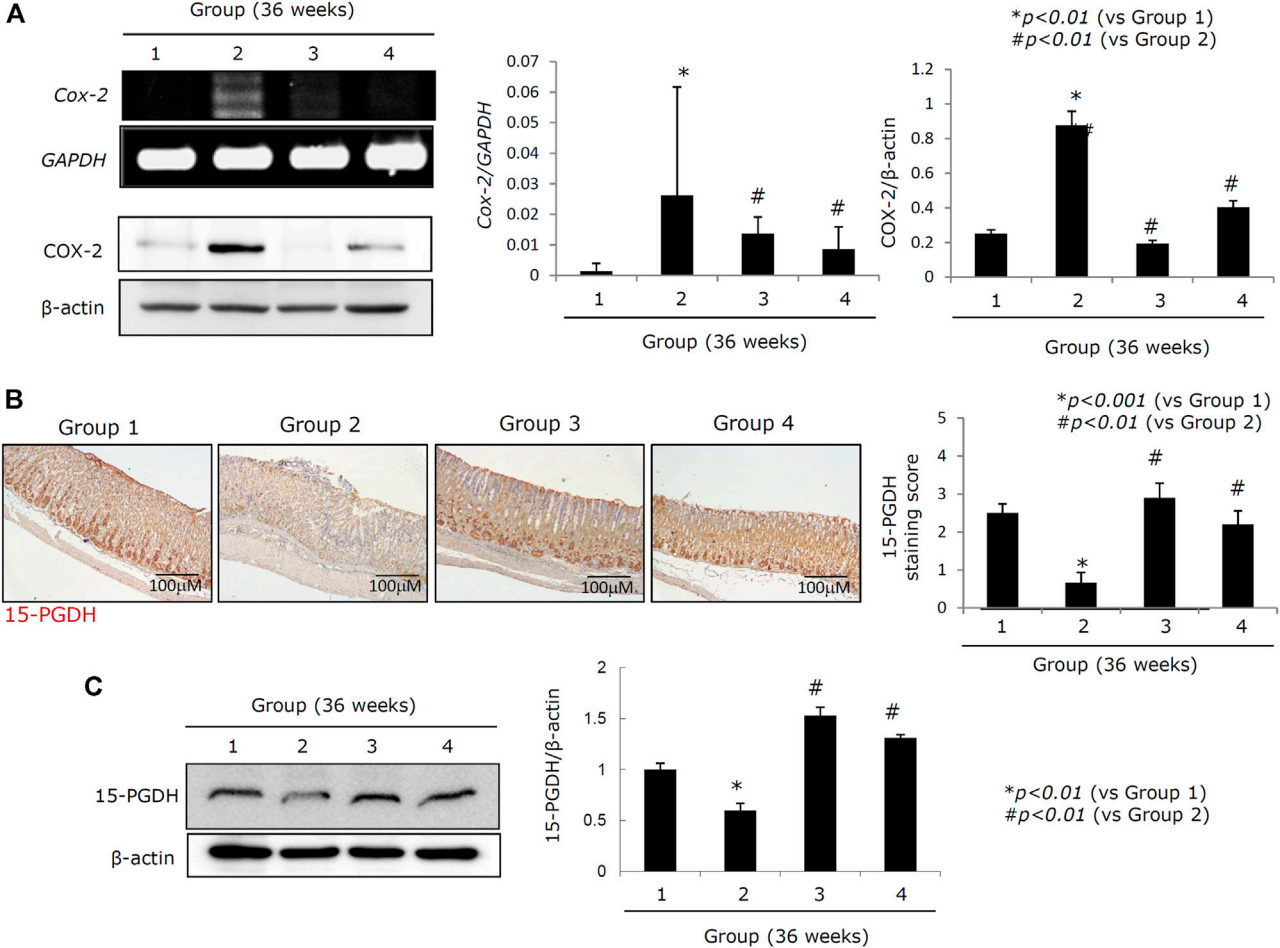
Changes of COX-2 and 15-PGDH according to groups. **(A)** RT-PCR for *COX-2* mRNA and western blot for COX-2. **(B)** Left: immunohistochemical staining for 15-PGDH, x100 magnification; right: mean expressions according to groups. **(C)** Western blot for 15-PGDH. All data represent mean ± SD (*n* = 10).

Increased expression of COX-2 is known to be responsible for perpetuated gastric inflammation and gastric carcinogenesis in chronic *H. pylori* infections (Resende et al., 2011; Thiel et al., 2011; Cheng and Fan, 2013; Echizen et al., 2016). When we measured *COX-2* mRNA and COX-2 protein expressions (Figure 4A), we noted a significant increase in both *COX-2* mRNA and COX-2 protein in Group 2 (*p < 0.001*). However, COX-2 expression was significantly decreased in both Groups 3 and 4 (*p < 0.01*, Figure 4A). COX induction can lead to an increase in 15-PGDH as part of the hormetic response designed to retain homeostasis. Expression of 15-PGDH is known to exert some tumor suppressive effects and has been linked to reducing tumorigenesis in response to *H. pylori*–mediated CAG. However, the mean expression of 15-PGDH in Group 2 was significantly lower than that in Group 1 (*p < 0.01;* Figure 4B), while 15-PGDH levels were significantly increased in Groups 3 and 4 compared to Group 2 (*p < 0.05*, Figure 4B). The results of the immunohistochemical staining of 15-PGDH were further confirmed by western blot against this protein in each group (*p < 0.01*, Figure 4C).

### PD-MSCs Exert an Anti-Inflammatory Effect Which Lowers Mutagenic Inflammation and Counteracts *H. pylori*–Induced Atrophy

**FIGURE 5:**
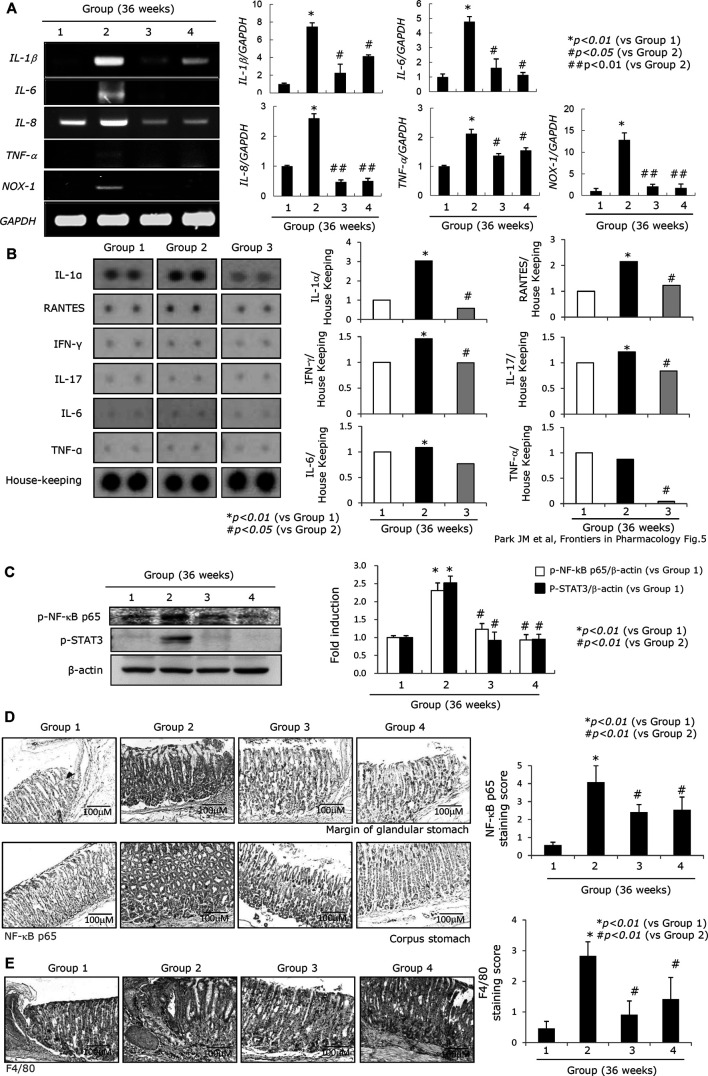
Changes of inflammatory mediators according to groups. **(A)** Left: RT-PCR for *IL-1β*, *IL-6*, *IL-8*, *TNF-α*, and *NOX-1* according to groups; right: mean expressions according to inflammatory genes. **(B)** Left: protein array for inflammatory proteins including IL-1α, RANTES, IFN-γ, IL-17, IL-6, and TNF-α; right: mean changes of individual inflammatory proteins on the protein array panel. **(C)** Western blot for p-NF-κB p65 and p-STAT3. **(D)** Immunohistochemical staining for NF-κB p65 according to groups. **(E)** Immunohistochemical staining for F4/80 denoting the status of macrophages according to groups, x100 magnification. All data represent mean ± SD (*n* = 10).

Oncogenic perpetuated gastric inflammation following *H. pylori* infection is the root cause of both CAG and gastric carcinogenesis (Piazuelo et al., 2010; Kim E.-H. et al., 2011). The expressions of inflammatory mediators such as IL-1β, IL-6, IL-8, TNF-α, and NOX-1 were all significantly increased in Group 2. However, administration of either the PD-MSCs or their concentrated CM led to a significant decrease in the expression of these inflammatory mediators (Figure 5). In this experiment, a protein array comprising several cytokines and chemokines was compared between the groups. The expressions of IL-1β, RANTES, IFN-γ, IL-17, IL-6, and TNF-α were all significantly increased in Group 2, but their expressions were all consistently attenuated in Group 3, signifying the contribution of the PD-MSCs to both the anti-inflammatory and anti-mutagenesis responses (Figure 5A). The RT-PCR evaluating the expression of the inflammatory mediators was then validated using a protein array experiment (Figure 5B). NF-κB activation and STAT3 phosphorylation are known to be involved in the progression of gastric inflammation following *H. pylori* infection. When we compare the expression of NF-κB and the phosphorylation of STAT3 between the groups (Figure 5C), we observed a significant increase in the activation of NF-κB κ and STAT3 in Group 2 and that the addition of PD-MSCs or their CM reversed these effects almost entirely. Infiltrating macrophages are the primary source of these inflammatory mediators following transcriptional activation, and we examined the expression of NF-κB, p65, and F4/80 through immunohistochemical staining. Figures 5C,D show that the highest expression levels of NF-κB and F4/80 (Figure 5E) were seen in Group 2 and that their expression was significantly decreased in Groups 3 and 4 (*p < 0.01*).

### Changes in the Activation and Expression of IL-10, IL-1β, and MMP Are All Critical in the Rejuvenation of CAG Tissues in Response to PD-MSC Treatment

**FIGURE 6:**
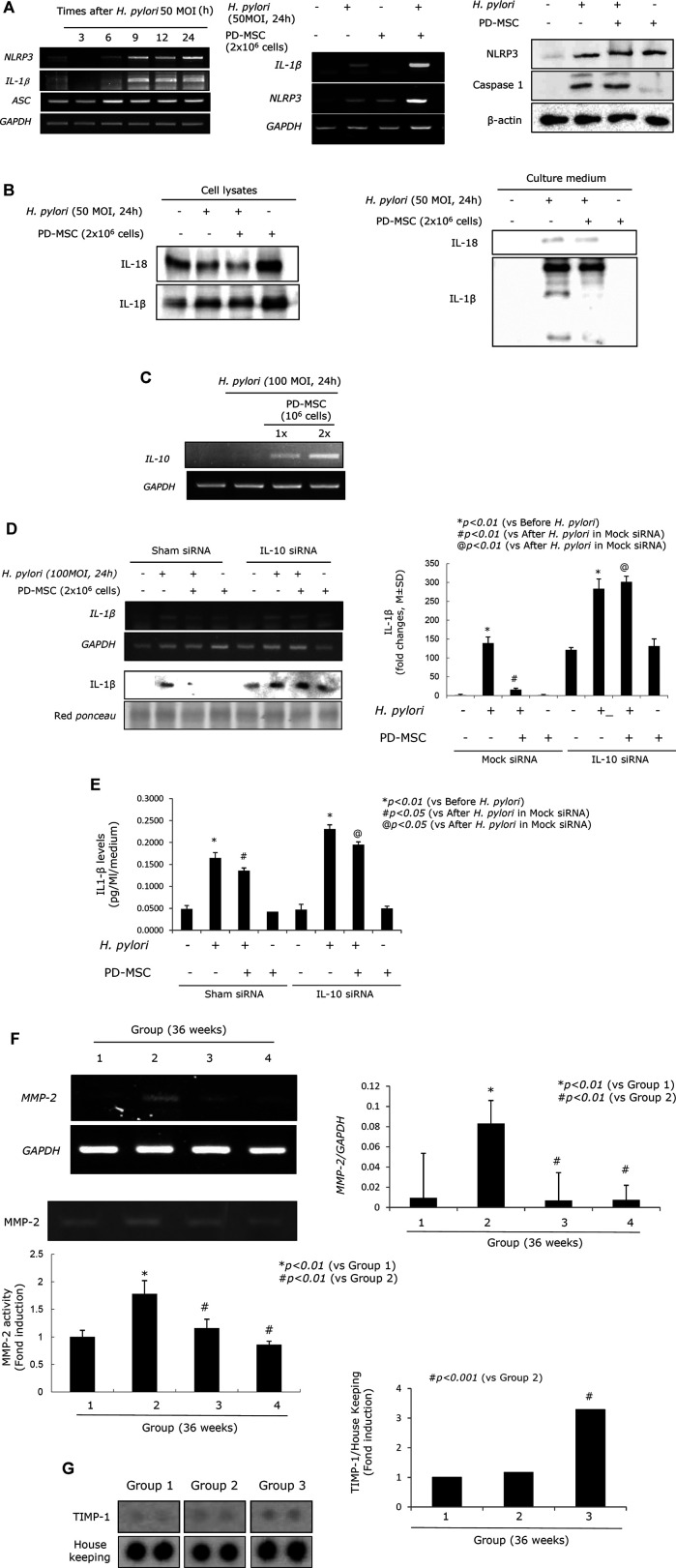
Inflammasomes relevant to *H. pylori* infection and PD-MSC influence. **(A)** Left: RT-PCR for *NLRP3*, *IL-1β*, and *ASC*. RT-PCR for *IL-1β* and *NLRP3* mRNA was repeated in the presence of PD-MSCs in RGM-1 cells using a transwell co-culture system. Right: western blot for NLRP3 and caspase-1 in the presence of PD-MSCs. **(B)** Western blot for IL-1β and IL-18 in cell lysate and cultured media in the presence of PD-MSCs. **(C)** IL-10 expressions according to PD-MSC RT-PCR for IL-10 mRNA. **(D)** IL-1β expression under *H. pylori* in the presence of PD-MSCs. **(E)** Left: IL-1β fold changes under *H. pylori* in the presence of PD-MSCs; right: IL-1β ELISA levels under *H. pylori* in the presence of PD-MSCs. **(F)** Upper: RT-PCR for *MMP-2*; lower: zymography for MMP-2. **(G)** Protein array for MMP-2. All data represent mean ± SD (*n* = 10).

Acute and chronic *H. pylori* infection leads to significant changes in atrophic gastritis (Correa and Piazuelo, 2008; Piazuelo et al., 2010; Correa and Piazuelo, 2011; Wroblewski et al., 2015), and *H. pylori* infection is defined as a class I carcinogen. Although eradication of *H. pylori* and non-anti-microbial interventions have been evaluated in the prevention of gastric cancer, the data suggest that the rejuvenation of precancerous atrophic gastritis seems to be the best way to prevent malignancy. Homeostasis seems to be very important in achieving rejuvenation in these tissues, and we hypothesize that IL-10 and the regulation of the inflammasome are critical to the success of therapeutic interventions using PD-MSCs. *H. pylori* infection is associated with increased inflammasome activity (Figure 6A; Supplementary Figure 4A) as *H. pylori* infection leads to increased NOD, LRR, and pyrin domain–containing protein 3 (NLRP3) and IL-1β expressions. PD-MSCs significantly increased inflammasome activation in the presence of *H. pylori* infection in the transwell co-culture system. However, *H. pylori* infection led to a significant decrease in IL-1β/IL-18 activity, while the administration of PD-MSCs in the presence of *H. pylori* infection led to significant inhibition of IL-18 and IL-1β secretion (Figure 6B; Supplementary Figure 4B). Under these conditions, IL-10 mRNA expression was significantly induced in response to PD-MSCs (Figure 6C; Supplementary Figure 4C), and where IL-10 induction was not feasible, the inhibitory action of PD-MSCs on IL-1β was significantly reduced (Figures 6D,E). This suggests that the significant anti-inflammatory actions of PD-MSCs were largely reliant on the concerted activity of various mechanisms for maintaining homeostasis. These actions were further supported by the significant inhibitory action of PD-MSCs on MMP, as seen in Figures 6F,G, which revealed a significant attenuation in *H. pylori*–induced MMP activity in response to these cells. Activated proteases, especially matrix metalloprotease (MMP), have been implicated in the propagation and aggravation of gastritis and the development of CAG or ulcers. This is supported by the fact that the expressions of MMP-2 and MMP-2 activity are significantly increased in Group 2 (*p < 0.001*). The protein array revealed that the expression of TIMP-1 was significantly increased in Group 3 (*p < 0.001*, Figure 6G), and these results were validated by a significant decrease in the expression of *MMP-2* mRNA and MMP-2 activity in the RT-PCR and zymography assays (Figure 6F).

### Changes in the Microbiome Reflect the Rejuvenation of *H. pylori*–Associated Atrophic Gastritis in Response to PD-MSC Treatment

**FIGURE 7:**
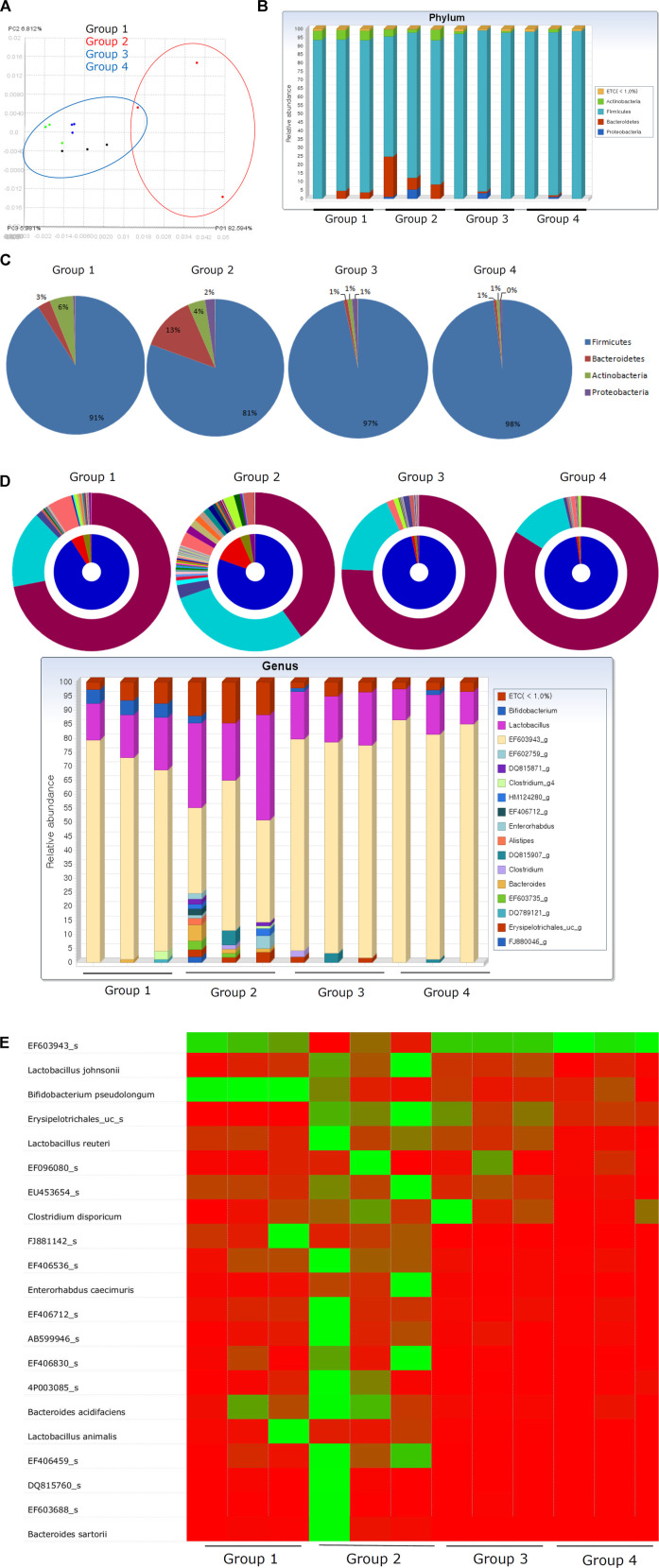
Fecal microbiota changes according to groups. **(A)** Principal coordinate analysis (PCoA) showing definite discrimination of microbiota according to PD-MSC administration, that is, between PD-MSC–treated and non-treated groups under *H. pylori*–induced CAG. **(B)** Phyla changes showing bar display. **(C)** Phyla level change. **(D)** Genus level changes with bar display. **(E)** Heatmap with microbiota nomination.

Changes in the microbiome in response to *H. pylori* infection are responsible for various gastric pathologies as bacterial overgrowth is closely associated with the changes in gastric atrophy and decreased gastric acidity (Huang et al., 2020; Lahner et al., 2020; Stewart et al., 2020; Ye et al., 2020). Although not clearly defined, overt changes in the intestinal microbiota do occur as the gastric pathology progresses toward atrophic gastritis. Figure 7A shows that the changes in the microbiota can be defined according to the group. Clear delineation was observed in PDA. Phylum analysis clearly showed that the phyla in Group 2 were quite different from those in Group 1 and Groups 3 and 4. Since the average gastric pathology was CAG, we speculated that the genera in Group 2 were also different from those in Group 1. However, Groups 3 and 4 showed a similar pattern to Group 1, suggesting that changes in CAG in Groups 3 and 4 might improve the community composition of the microbiome in these mice and facilitate their return to a more normal profile (Figure 7B,C). A detailed analysis of the genera in these samples (Figure 7D) revealed significant changes in the gastric microbiota in Group 2, but not in Groups 3 and 4 when compared to Group 1. The heatmap in Figure 7E and Supplementary Table 3 summarize the detailed changes in the gastric microbiota of these animals. We concluded that the administration of PD-MSCs and their CM significantly rejuvenated *H. pylori*–associated CAG, leading to the expectation that MSCs can be used as potential cell therapeutics to reverse precancerous atrophic changes after chronic *H. pylori* infection.

## Discussion

This study sheds light on the potential of using PD-MSCs or their CM as therapeutics to rejuvenate precancerous atrophic gastritis in order to reduce pathogenic progression. In addition to the basic proliferative, restorative, anti-inflammatory, immunomodulatory, and regenerative effects of the MSCs, treatment with these agents induces the expression of LGR5+ (Mills and Shivdasani, 2011; Ye et al., 2018) and Musashi-1 (Murata et al., 2008) and promotes Ki-67–mediated proliferation (Kim et al., 2004) and anti-apoptotic effects while reducing NF-κB expression (Ralhan et al., 2009) and inhibiting oncogenic STAT3 expression (Balic et al., 2020). The application of PD-MSCs also promotes the cross-talk between the inflammasome and autophagy pathways and 15-PGDH in a chronic *H. pylori* infection model (Figure 8).

PD-MSCs have multiple properties, including strong self-renewal, multi-potent differentiation, immunomodulatory, anti-inflammatory, antioxidative, and regenerative capabilities (Kim M. J. et al., 2011; Seok et al., 2020a; Chou and Chen, 2020; Saleh et al., 2020; Yuan et al., 2020). Among the various types of MSCs, including umbilical cord–derived MSCs, chorionic plate–derived MSCs, adipose-derived MSCs, and bone marrow–derived MSCs, PD-MSCs are best known for their secretion of various cytokines, including growth factors such as G-CSF; regulated upon activation, normal T cell expressed and secreted (RANTES); and immunomodulators such as IL-6, IL-8, and IL-10, and have been linked to the effective treatment of various degenerative and destructive diseases (Lee et al., 2010; Lee et al., 2012; Jung et al., 2013; Munir et al., 2019; Seok et al., 2020b). The reasons why the MSCs have emerged among the most promising regenerative tools are closely linked to their multi-differentiation potential and immunosuppressive capacity. PD-MSCs are preferred within the MSC cohort because of their superior proliferation capacity, lower immunogenicity, and likely lower mutation rates than other kinds of MSCs originating from the amniotic membrane (AM), umbilical cord (UC), *decidua parietalis*, and chorionic plate (CP) (Wu et al., 2018; Chen et al., 2019; Guan et al., 2019; Ma et al., 2019).

Robust apoptosis after *H. pylori* infection is one of the core mechanisms responsible for atrophic gastritis (Figures 3A,B). Multiple studies have attempted to clarify the related autophagy mechanisms underlying apoptosis, clearing of damaged organelles, cell debris, and external pathogens needed to maintain the genomic integrity of cells, supply more energy, maintain cell or tissue homeostasis, inhibit endoplasmic reticulum (ER) stress, maintain ER function by degrading unfolded protein aggregates, and promote cell growth and proliferation in response to CAG (Lum et al., 2005; Karantza-Wadsworth et al., 2007; Hu et al., 2020). In Figures 3E–G, we clearly document the contribution of PD-MSCs to the protective actions of induced autophagy as part of relieving *H. pylori*–associated CAG for the first time, although multiple reports have revealed modulating autophagy as a primary mechanism in the protective effects of MSCs used to prevent hypoxia and ischemia or infection-induced post-injury toxicity in affected organs (Golpanian et al., 2016; Hu and Li, 2018; Hu et al., 2019; Zhang et al., 2019). Although the impacts of the autophagic processes are different, that is, protective against *H. pylori*, determining the intracellular fate of *H. pylori*, and carcinogenic in infected cells (Wang et al., 2009; Chu et al., 2010; Deen et al., 2013; Pott et al., 2018), our study clearly shows the therapeutic potential of autophagic processing of PD-MSCs at the CAG stage of gastric cancer lesion development.

In this study, PD-MSCs exerted a significant rejuvenating effect against *H. pylori*–induced atrophic gastritis by regulating the inflammasomes associated with autophagy induction and inducing a significant increase in anti-inflammatory IL-10 production (Figure 6). Cross-talk between the inflammasomes and the autophagy pathways plays an important role in intracellular homeostasis, inflammation, immunity, and pathology, after which the dysregulation of these processes is often associated with the pathogenesis of numerous cancers, including *H. pylori*–associated pro-tumor and gastric cancer (Chung et al., 2020). Inflammasomes are multi-protein complexes that assemble in the cytosol of cells upon detection of pathogen- or danger-associated molecular patterns (PAMP/DAMP) (Broz and Monack, 2011; Russo et al., 2018; Seveau et al., 2018). A critical outcome of inflammasome assembly is the activation of serine protease caspase-1, which activates the pro-inflammatory cytokine precursors pro-IL-1β and pro-IL-18, as shown in Figure 6. However, in the presence of *H. pylori* infection, PD-MSCs significantly inhibited secretion of IL-1β via their active secretion of anti-inflammatory IL-10.

The question of whether the reversal of gastric atrophy following *H. pylori* eradication is possible was not answered before 1998 (Domellof, 1998), but extensive evaluations, research, and nationwide trials in Japan have shown that it may be possible to repair the damage associated with *H. pylori* infection and prevent gastric cancer (Sugano et al., 2015; Tsuda et al., 2017; Choi et al., 2018; Choi et al., 2020). Furthermore, the non-microbial approach including the application of phytochemicals, probiotics, *n*-3 polyunsaturated fatty acids (*n*-3 PUFAs), walnut, and fermented kimchi (Chung and Hahm, 2010; Kim et al., 2010; Jeong et al., 2015; Lee et al., 2015; Park et al., 2015; Han et al., 2016; Jeong et al., 2016) can rejuvenate CAG. Given this success, this study was designed to evaluate the application of stem cells or their conditioned medium as candidates for clinically relevant therapeutic intervention in CAG and the downstream prevention of gastric cancer. We tried to apply stem cells at the atrophic gastritis stage as the above non-microbial approaches were usually implemented before CAG development and independent from *H. pylori* eradication (Oh et al., 2006; Giannakis et al., 2008; Kim, 2019).

Moreover, in this study, we reported that PD-MSCs induced more Lgr5+ cells, thereby facilitating the recovery from CAG induced by *H. pylori*. A stem cell niche includes both Wnt and BMP signaling pathways, and the balance between their signaling is important within the intestine. In our recent study, the Wnt signaling pathway has emerged as a potential regulator of self-renewal for intestinal stem cells by PD-MSCs (Han et al., 2017). Furthermore, induction of Wnt/β-catenin and growth factor signaling rescues liver dysfunction through the induction of Lgr5+ cells (Lin et al., 2017). PD-MSCs in the current study faithfully contributed to the regeneration of the ulcerated tissue structure. PD-MSCs effectively enhanced or maintained the regeneration process along with significantly concerted actions of anti-inflammation, anti-apoptosis, and induction of autophagy and inflammasomes.

Lastly, this study revealed several interesting observations related to the fecal microbiome and its ability to reflect improvements in CAG associated with PD-MSC treatment. Figure 7 clearly shows the changes in the distribution of the phyla and genera in each group, with significant changes in the composition of the microbiota noted in Group 2 when compared with that in Group 1, signifying a relationship between this microbiome and atrophic changes in the gastric tissues. However, these microbiome changes could be reversed following the application of either the PD-MSCs or their CM. Detailed microbiomes are presented in Supplementary Table 3 and support our conclusion that PD-MSCs afforded some repair to the microenvironment producing one that is more favorable to supporting non-atrophic conditions. Although the exact changes in the gastric microbiome across stages of neoplastic progression remain poorly understood, the study by Wang *et al.*(Wang Z. et al., 2020) showed that the bacterial diversity and abundance of Armatimonadetes, Chloroflexi, Elusimicrobia, Nitrospirae, Planctomycetes, Verrucomicrobia, and WS3 decrease as atrophy progresses and Actinobacteria, *Bacteroides*, Firmicutes, Fusobacteria, SR1, and TM7 were enriched in the intestinal metaplasia (Coker et al., 2018; Park et al., 2019; Yu et al., 2020). The results of our fecal microbiota analysis according to therapeutic intervention to rejuvenate CAG increased the potential MSCs or their CM as therapeutics, similar to cosmetics for aged skin. Among the microbes in the stomach, *H. pylori* remains the single most important risk factor for gastric disease, its capacity to shape the collective gastric microbiota as well as its contribution to pathogenesis should be further elucidated (Rajilic-Stojanovic et al., 2020), and current results suggest a definite role for this pathogen in CAG since the addition of PD-MSCs significantly changed the fecal microbiome.

In this study, we have shown that oral administration of PD-MSCs or their CM rejuvenated the *H. pylori*–associated CAG by stimulation of gastric stem cells, induction of autophagy, inhibition of inflammation, and induction of inflammasomes. As a non-microbial approach for *H. pylori*–associated CAG, supplementation or treatment with long-term phytochemicals, antioxidants, and probiotics was proven to be very efficacious in the prevention of *H. pylori*–associated CAG and carcinogenesis. These treatment strategies were supported by anti-inflammation and cytoprotection activities by targeting small molecules or regulating signaling cascades. Although MSC administration has a significant inhibitory effect on the inflammatory response, there remain problems in clinical trials of stem cell therapy, including embolism risk and immunogenic adverse effect. Therefore, a lot of studies have been conducted to know the effect and underlying mechanism of MSC–CM on inflammation-related diseases. Factors secreted by MSC have been reported to include inflammatory modulators such as transforming growth factor-β (TGF-β), TNF-stimulated gene-6 (TSG-6), prostaglandin E2 (PGE2), and hepatocyte growth factor (HGF). However, to the best of our knowledge, the effect of oral administration of MSCs and CM on inflammatory diseases has not yet been reported. The data obtained suggested the use of MSCs and CM to treat inflammatory diseases without any cell transplantation.

In conclusion, this study shows that the administration of PD-MSCs or their CM at the CAG stage can produce a significant rejuvenation of the gastric tissues and help to prevent or revert the *H. pylori*–induced pro-tumor conditions in these tissues. Our evaluations revealed several novel mechanisms facilitating these effects which add to the basic proliferative, self-renewal, and regenerative capability of these cells. PD-MSCs exerted inflammasomes/autophagy/15-PGDH induction/IL-10 induction (Figure 8). The next step is to evaluate these effects in careful clinical trials.

## Data Availability

The datasets presented in this study can be found in online repositories. The names of the repository/repositories and accession number(s) can be found in the article/Supplementary Material.
