# Supplementary figures and images for: Rejuvenation of Helicobacter pylori–Associated Atrophic Gastritis Through Concerted Actions of Placenta-Derived Mesenchymal Stem Cells Prevented Gastric Cancer
Source: Front Pharmacol. 2021 Aug 18;12:675443. doi: 10.3389/fphar.2021.675443 (PMC8416416; doi:10.3389/fphar.2021.675443)

(A)

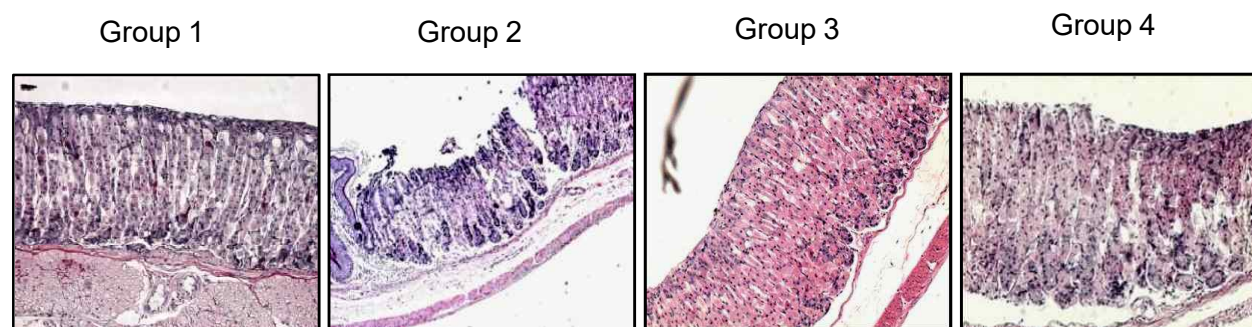

(B)

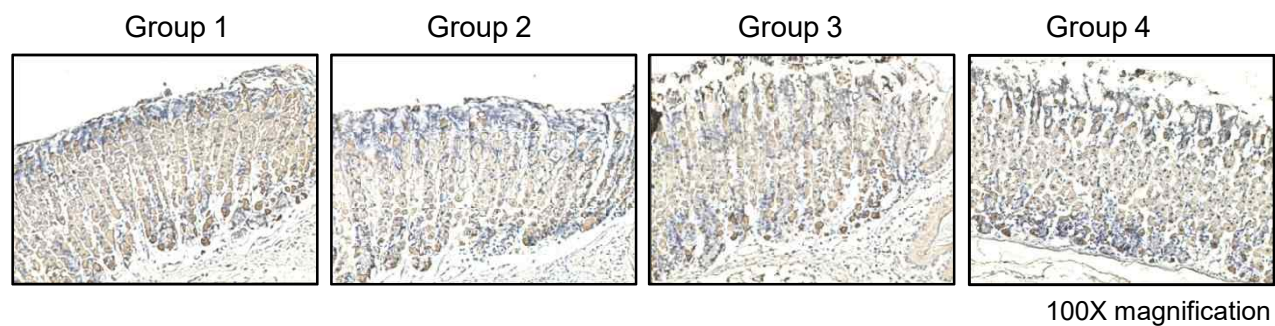

(C)

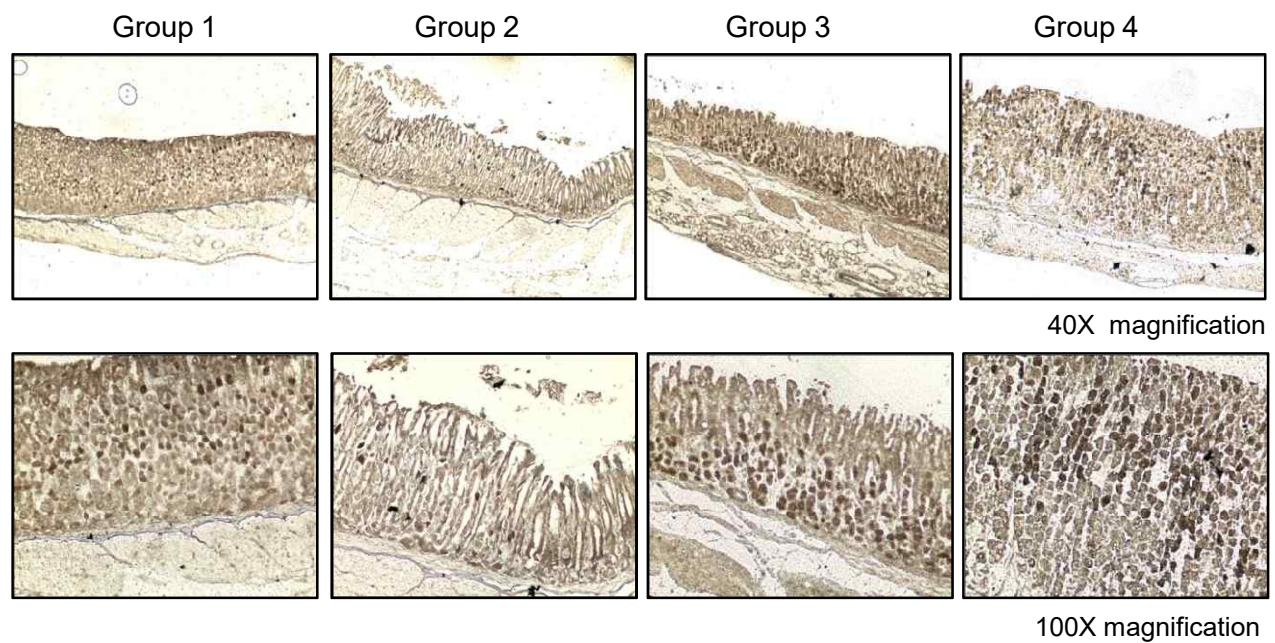

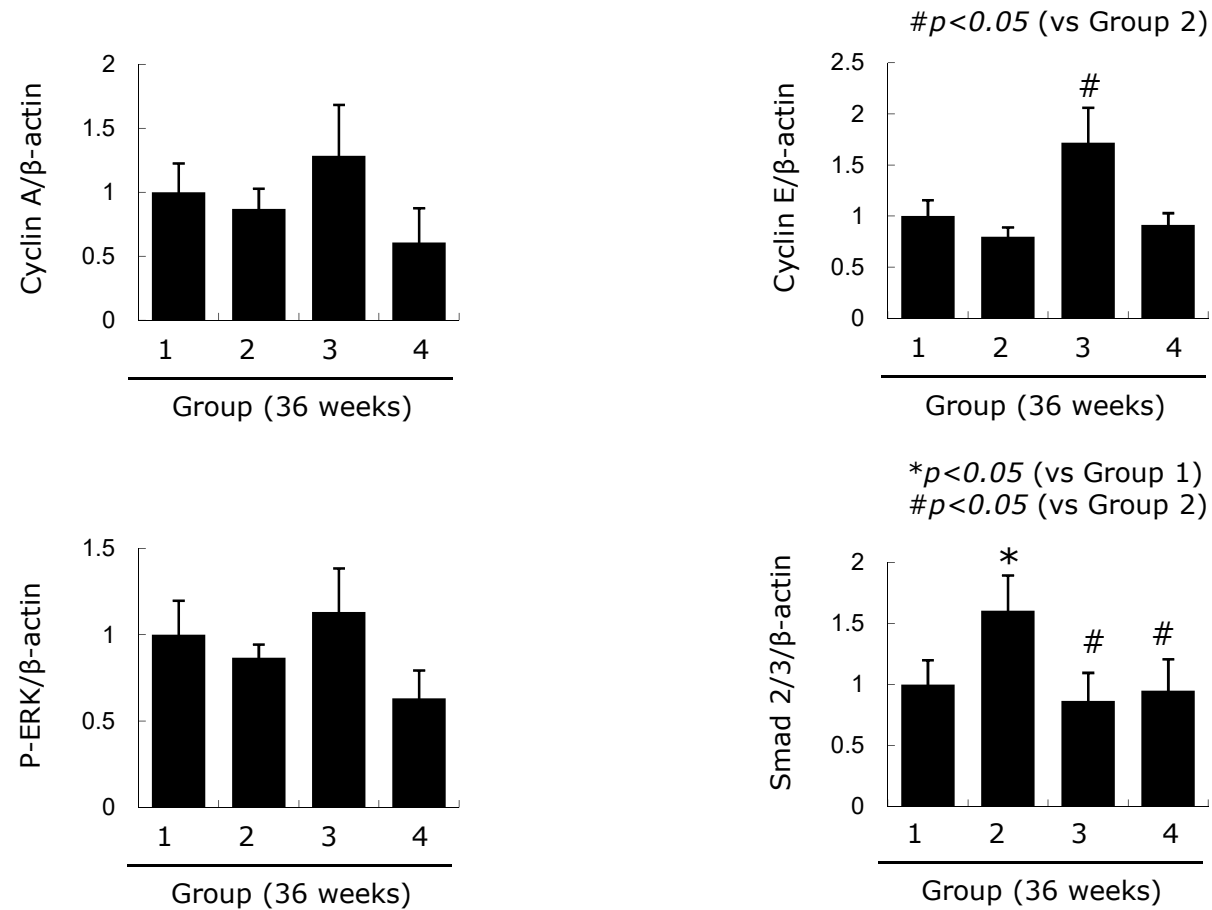

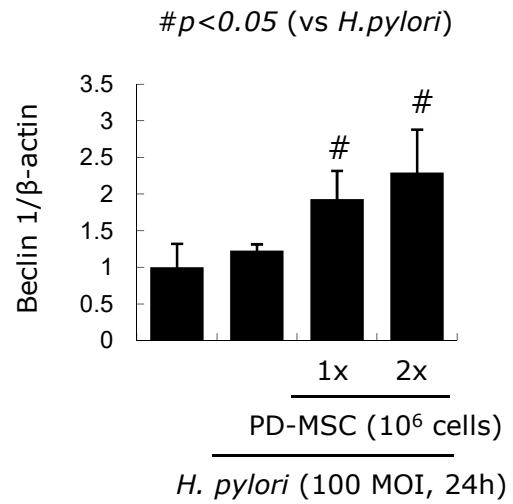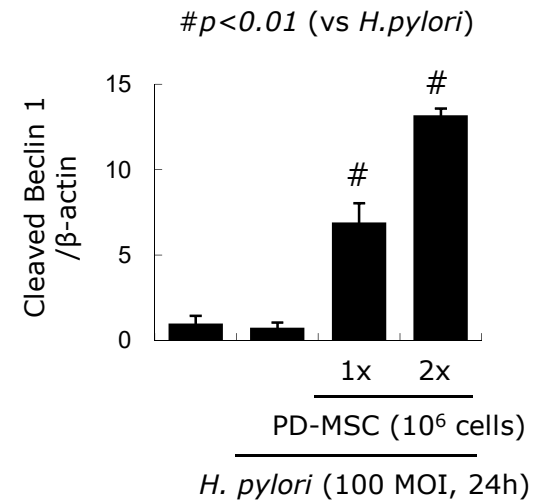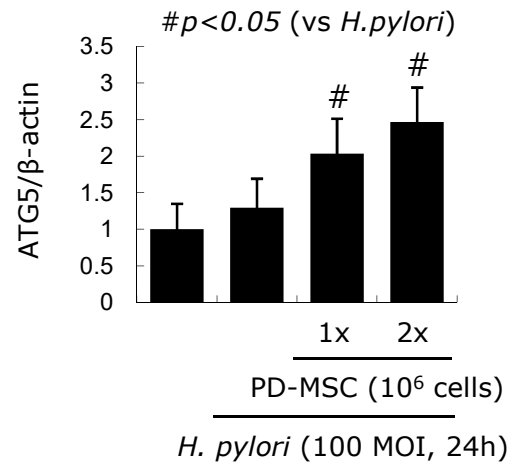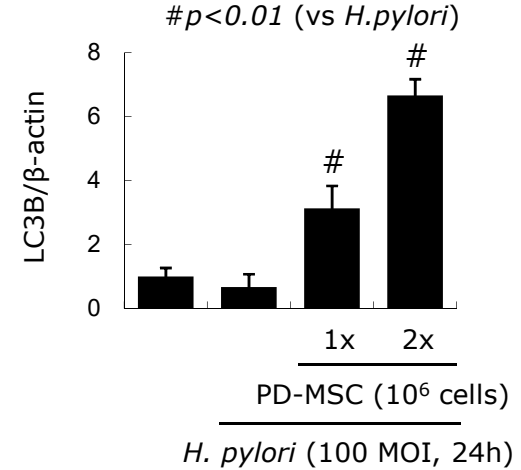

(A)

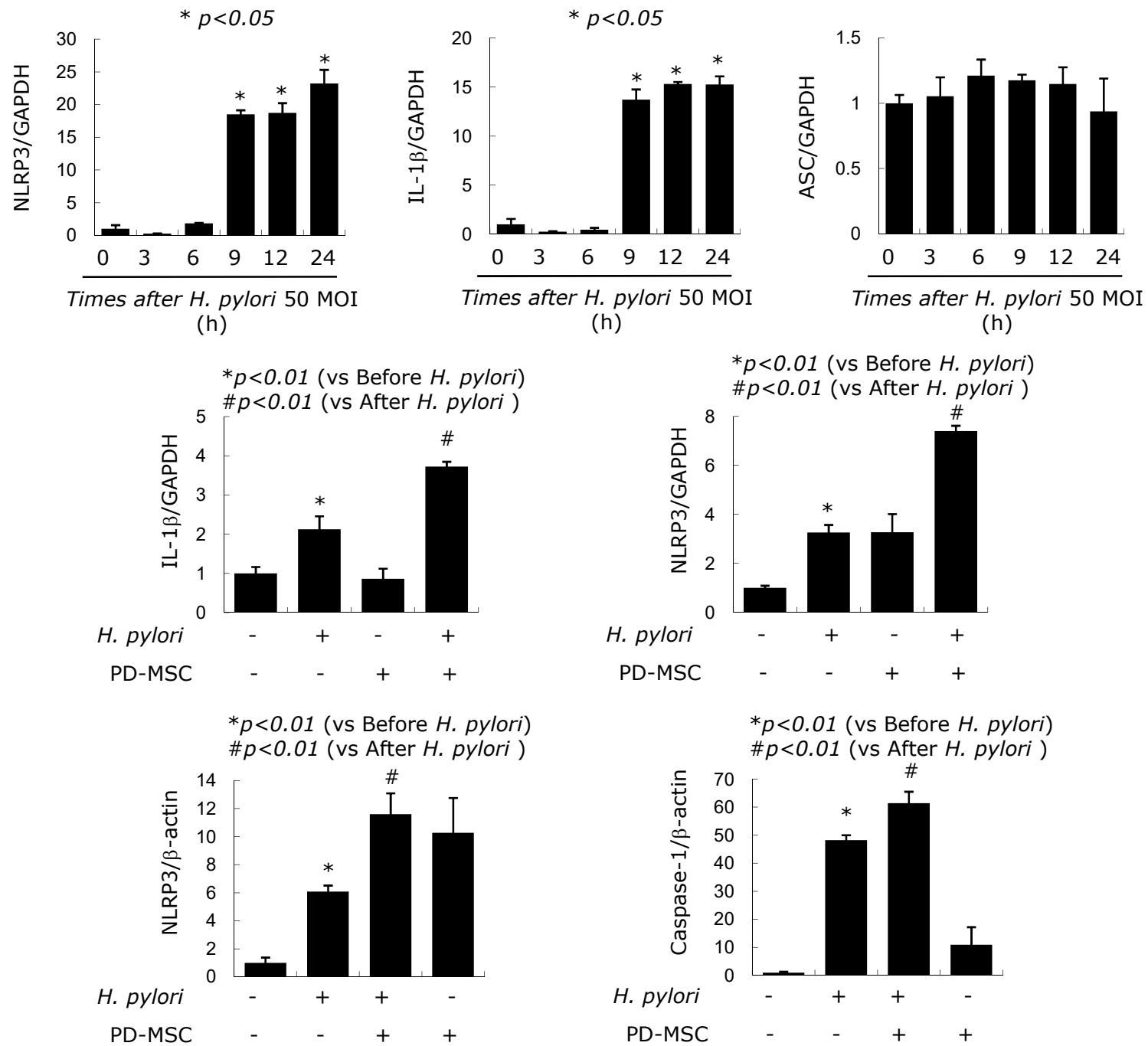

(B)

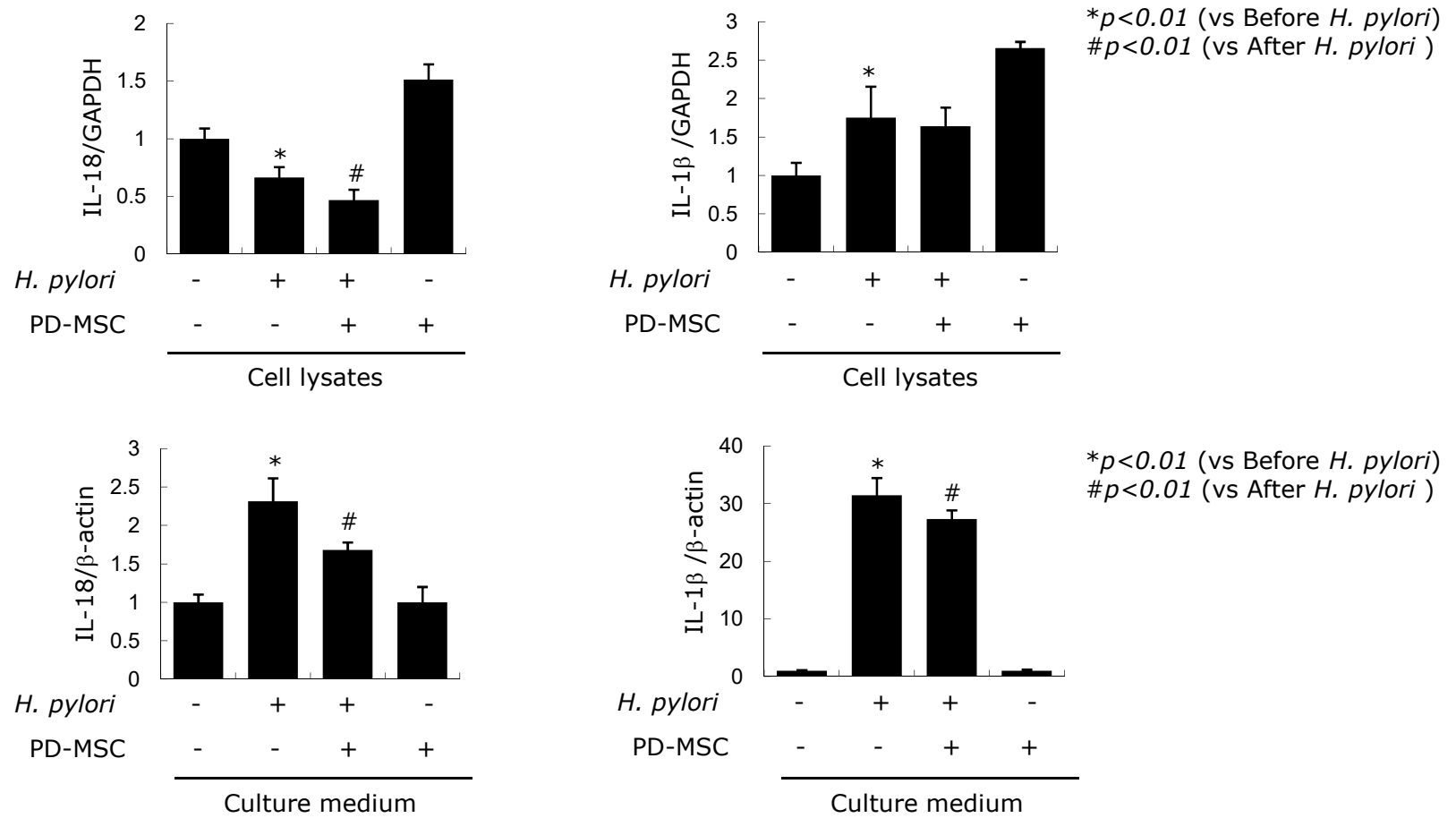

(C)

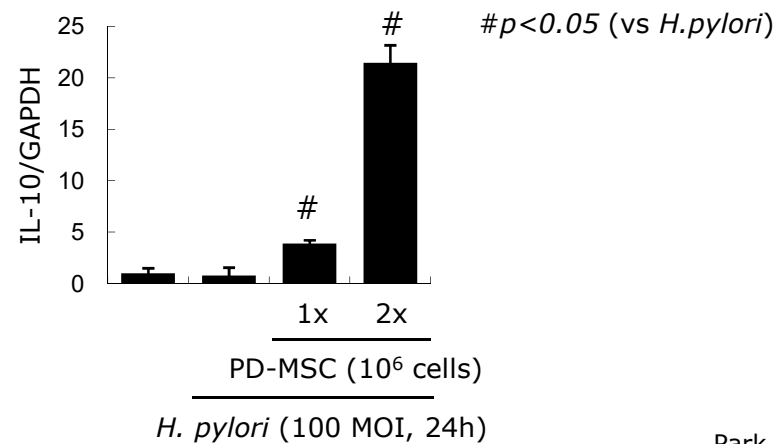

Supplement: Supplementary file 1 [file DataSheet2.PDF]
