# Supplementary material for: Rejuvenation of Helicobacter pylori–Associated Atrophic Gastritis Through Concerted Actions of Placenta-Derived Mesenchymal Stem Cells Prevented Gastric Cancer
Source: Front Pharmacol. 2021 Aug 18;12:675443. doi: 10.3389/fphar.2021.675443 (PMC8416416; doi:10.3389/fphar.2021.675443)
Supplement: Supplementary file 2 [file DataSheet1.DOCX]

**Supplementary information**

**Gross lesion index**

After sacrificing the mice, the isolated stomachs were open along the greater curvature and washed in ice-cold saline. To investigate the degree of gross mucosal pathology, the mucosal sides of the stomachs were photographed using a digital camera and part of the mucosa was immediately fixed with 10% formalin solution. The gross damage of the gastric mucosa was assessed by three gastroenterologists, who were blinded to the treatments, using a gross ulcer index [1].

**Index of histopathologic injury**

For histopathological analysis, the stomach was fixed in 10% neutralized buffered formalin, processing using the standard method and embedded in paraffin. Sections of 4 μm thickness were then stained with hematoxylin and eosin. The glandular mucosa of corpus and antrum were examined histologically. The pathological changes of H*. pylori*-infection, such as inflammatory cells infiltration, erosive lesions, ulceration, dysplasia, adenoma formation (precancerous lesion), were graded by three gastroenterologists, who were blinded to the group, using an index of histologic injury defined. In this study, inflammation was defined as grade the infiltration of inflammatory cells, 0: none, 1: under the lamina propria, 2: half of mucosa 3: until the epithelial gland layer (all mucosa). The erosion was defined as proportion of erosive lesion, 0: none, 1: loss of epithelial gland layer (1/3 proportion), 2: two-three portion of mucosa (2/3 proportion) 3: all mucosa (3/3 proportion)

**Immunohistochemical staining**

Immunohistochemistry was performed on replicate sections of mouse gastric tissues. After deparaffinization were dewaxed and rehydrated with graded alcohol, and boiled three times in 100mM Tris buffered saline (pH 6) with 5% urea in an 850W microwave oven for 5min each. And then cooling in water for 15 min and washed in PBS, and slides were incubated overnight with the primary antibody at 4°C. Antibodies:F4/80 (1:500; eBioscience, San Diego, CA) or 15-PGDH (1:300; Dako, Santa Clara, CA) or Ki-67 (1:300; Santa Cruz, Santa Cruz, CA) in the presence of 1.0% bovine serum albumin respectively. Slides incubated with secondary antibody (1:300) for 1hr at room temperature, and then with 4-6- diamidino-2-phenylindole (DAPI, 100 ng/ml) for 1 min at room temperature. Finally, the slides were counterstained with hematoxylin (Sigma-Aldrich). After incubation, a subsequent reaction was formed using a Vector kit (Vector Laboratories, Inc., Burlingame, CA). Finally, the slides were incubated with 3, 3’-diaminobenzidine (Invitrogen Life Technologies, Carlsbad, CA) and counterstained with hematoxylin (Sigma-Aldrich, St. Louis, MO).

**Terminal deoxynucleotidyl transferase-mediated dUTP nick-end labeling (TUNEL) staining**

Apoptosis was visualized using a terminal deoxynucleotidyl transferase (TdT) fRAGel DNA fragmentation detection kit (Oncogene Research Products, La Jolla, CA). To determine the apoptotic index in each group, TUNEL immunostained sections were scanned under low-power magnification (×100) to locate the apoptotic hotspots.

**RT-PCR**

Total RNA was isolated using the Trizol (Invitrogen, Carlsbad, CA). Trizol was added to 1.5ml tube, which were then incubated 10 min at 4°C and gently mixed with 100µl chloroform (Merck, Rahway, NJ). After incubation for 10 min in ice, samples were centrifuged at 10,000 x *g* for 30 min. Supernatants were extracted and mixed with 200 µl isopropanol (Merck), and mixtures were incubated at 4°C for 1 h. After centrifuging at 13,000 *g* for 30 min, pellets were washed with 70% (*v*/*v*) ethanol. After allowing the ethanol to evaporate completely, pellets were dissolved in 40µl diethylene pyrocarbonate-treated water (Invitrogen Life Technologies). cDNA was prepared using reverse transcriptase originating from Murine-Moloney leukemia virus (Promega, Madison, WI), according to the manufacturer’s instructions. The polymerase chain reaction (PCR) was performed over 25 cycles of 94°C for 20 s, 58.5 for 30 s, and 72°C for 45 s. Oligonucleotide primers were purchased from Bioneer (Daejeon, Korea). Oligonucleotide primers were as follows; for PDGF, sense 5'-AGG AAG CCA TTC CCG CAG TT-3' and antisense 5'-CTA ACC TCA CCT GGA CCT CT-3', for FGF, sense 5'-GAG GAG TTG TGT CTA TCA AAG-3' and antisense 5'-GTT CGT TTC AGT GCC ACA TAC C-3', for HGF, sense 5'-ATC CAC GAT GTT CAT GAG AG-3' and antisense 5'-GCT GAC TGC ATT TCT CAT TC-3', for COX-2, sense 5'-CAT CCT GCC AGC TCC ACC GC-3' and antisense 5'-GGG AGG AAG GGC CCT GGT GT-3', for 15-PGDH, sense 5'-CAG GCA GAG AAT GCT GAG TTC-3' and antisense 5'-GAT GTT GAG CAG GAA CGC AGT-3', for IL-1β, sense 5'-CAG GCT CCG AGA TGA ACA ACA AAA-3' and antisense 5'-TGG GGA ACT CTG CAG ACT CAA ACT-3', for IL-6, sense 5'-GGG ACT GAT GCT GGT GAC AA-3' and antisense 5'-TAA CGC ACT AGG TTT GCC GA-3', for IL-8, sense 5'-GTG GCT TTG CCG TGC AAT AA-3' and antisense 5'-GCA CAG GGT TGA GCC AAA AG-3', for TNF-α, sense 5'-TTC TAT GGC CCA GAC CCT CA-3' and antisense 5'-CTC CAA AGT AGA CCT GCC CG-3', for NOX-1, sense 5'-GGG ATG ACC ATA AGG GGA GT-3' and antisense 5'-CCC AAC CAG TAC AGC CAC TT-3', for MMP-2, sense 5'-GAG TAA GGG GAT CGC CGT GCA-3' and antisense 5'-AAG AGG TTG CAA CTC TCC TTG G-3', for NLRP3, sense 5'-GGT CCT CTT TAC CAT GTG CTT C-3' and antisense 5'-AAG TCA TGT GGC TGA AGC TGT A-3', for IL-1β (for RGM-1 cells), sense 5'-ACC TAT GTC TTG CCC GTG GA-3' and antisense 5'-GTG GGT GTG CCG TCT TTC AT-3', for ASC, sense 5'-GCA ACT GCG AGA AGG CTA TG-3' and antisense 5'-AAG CAT CCA GCA CTC CGT C-3', for IL-10, sense 5'-GGG TTA CTT GGG TTG CCA AG-3' and antisense 5'-GTC CTG CAG TCC AGG ATA TG-3', and for *GAPDH*, sense 5'-AAT GTA TCC GTT GTG GAT CT-3' and antisense 5'-TCC ACC ACC CTG TTG CTG TA-3'.

**Western blotting**

Cells or resected gastric tissues were harvested and lysed in lysis buffer (Cell signaling Technology) containing 1mM phenylmethylsulfonyl fluoride (PMSF, Sigma Aldrich St. Louis, MO). After 30min of incubation, samples were centrifuged at 12,000 x *g* for 15min 4°C. The supernatants were then collected and protein quantification was carried out with a Bio-Rad protein assay. Equal amounts soluble protein (30μg) were denaturated by heating at 100°C for 3minutes. Proteins were separated by sodium dodecyl sulphate-polyacrylamide gel electrophoresis (SDS-PAGE) and transferred to polyvinylidene fluoride membranes. The membranes were blocked in 5% BSA in PBST for 30min. And then, the membranes probed initially with specific primary antibody, washed, incubated with peroxidase-conjugated secondary antibodies, and rewashed. The protein bands were detected by chemiluminescence (Supersignal, Pierce) exposure on chemiluminescence system(GE Healthcare, Buckinghamshire, UK).The general procedure for Western blot analysis of cultured mouse gastric mucosal cells was similar to the procedures described above. Antibodies used in the current study were COX-2, purchased from Thermo, β-actin purchased from Santa Cruz, 15-PGDH, purchased from Cayman. Primary antibody against actin was purchased from Sigma-Aldrich Co. (St. Louis, MO), antibodies for lamin B from Santa Cruz Biotechnology (Santa Cruz, CA), other antibodies for p-STAT3^Tyr705^, total STAT3from Cell Signaling Technology (Beverly, MA), horseradish peroxidase-conjugated secondary antibody from Pierce Biotechnology (Rockford, IL). DL-dithiothreitol (DTT), TRIzol^®^, 4',6-diamidino-2-phenylindole (DAPI)from Invitrogen (Carlsbad, CA), and polyvinylidene difluoride (PVDF) membranes were supplied from Gelman laboratory (Ann Arbor, MI). The ECL chemiluminescent detection kit was purchased from LPS solution (Daejon, South Korea) and protein assay dye (Bradford) reagent was supplied by Bio-Rad Laboratories (Hercules, CA), bicinchonic acid (BCA) protein assay reagent was obtained from PierceBiotechnology (Rockford, IL). β-actin, Lamin B, cyclooxygenase (COX-2), nitric oxide synthase (iNOS), p53, cytochrome c, surviving antibodies were purchased from Santa Cruz Biotechnology (Dallas, TX), phosphorylated signal transducer and activator of transcription 3 (STAT3), Bax, B-cell lymphoma 2 (Bcl-2), cleaved caspase-3, cleaved caspase-8, poly-ADP-ribosepolymerase (PARP), and Musashi-1 from Cell Signaling Technology (Danvers, MA).

**Cytokine protein array**

Cytokine protein array was performed using Mouse Cytokine Antibody Array 3 (4 membrane arrays) with Accessories, for simultaneous detection of 54 cytokines related proteins in 2 samples from R&D systems (Minneapolis, MN). After blocking the array membranes for 30 min, the membranes were incubated with 1ml of serum at room temperature for 2 hr. After washing with buffer, we added primary biotin-conjugated antibody to each membrane, for incubation at room temperature for 2 hr. After washing with buffer and addition of horseradish peroxidase-conjugated streptavidin to each membrane, we exposed them to detection buffer, using a luminescent image analyzer system (LAS-4000, Fuji Film, Tokyo, Japan). Density was expressed as the percentage of the detected value from the sample versus the background result, using a gelpro32 program (Media Cybernetics, Rockville, MD).

**Preparation of cytosolic and nuclear extracts**

After *H. pylori* infection, resected stomach tissues were washed twice with ice-cold 1x PBS and scraped in 1 mL of PBS, followed by centrifugation at 1,700x*g* for 5 min at 4°C. Pellets were resuspended in hypotonic buffer A [10 mM N-2-hydroxyethylpiperazine-N’-2-ethanesulfonic acid (pH 7.9), 1.5 mM MgCl_2_, 10 mM KCl, 0.5 mM DTT and 0.2 mM phenylmethylsulfonylfluoride (PMSF)] for 15 min on ice. Ten % Nonidet P-40 was then added to final concentration of 0.1% for less 3than 5 min. The mixture was then centrifuged at 6,000 x *g* for 5 min at 4°C. Supernatant was collected as the cytosolic extract and stored at -80°C. The pellets were washed twice with hypotonic buffer A and resuspended again in hypertonic buffer C [20 mM N-2-hydroxyethylpiperazine-N’-2-ethanesulfonic acid (pH 7.9), 20% glycerol, 420 mM NaCl, 1.5 mM MgCl_2_, 0.2 mM ethylenediaminetetraacetic acid, 0.5 mM DTT and 0.2 mM PMSF] for 1 h on ice and centrifuged at 18,000 x *g* for 15 min at 4°C. The supernatant containing nuclear proteins was collected and stored at -80°C. The protein concentrations of both fractions were determined by using the BCA protein assay reagent.

**RNA interference**

The expression of LC3B and IL-10 were abolished using target-specific small interference RNA (siRNA) molecules purchased from Bioneer (Daejeon, Korea). LC3B and IL-10 siRNA were transfected into RGM1 cells using Lipofectamine reagent (Invitrogen, Carlsbad, CA) according to the manufacturer’s instructions.

**Zymography**

For the zymography assay, cells treatment were performed without serum. Enzymatic activity of MMPs -2 was assayed by gelatin zymography. Samples were electrophoresed on gelatin-containing 10% SDS-polyacrylamide gels. The gel was washed twice with washing buffer (50 mM Tris–HCl, pH 7.5, 100 mM NaCl and 2.5% Triton X-100), followed by brief rinsing in washing buffer without Triton X-100. This was followed by treatment with incubation buffer (50 mM Tris–HCl, pH7.5, 150 mM NaCl, 10 mM CaCl2, 0.02% NaN3 and 1 μM ZnCl2) at 37 °C. Next, the gel was stained with Coomassie brilliant blue R-250 (Sigma, St Louis, MO), and destained. A clear zone appearing on the gel signified the presence of MMP.

**Bacteria DNA extraction from mouse stool samples**

The stool sample is filtered through a 40 μm-pore sized cell strainer after being diluted and incubated in 10mL of PBS for 24 hr. To separate the bacteria from human stool, bacteria in stool samples is isolated using centrifugation at 10,000 x *g* for 10 min at 4℃. After centrifugation, the pellet is comprised of bacteria. To extract the DNA out of the bacteria, bacteria is boiled for 40 min under 100℃. DNA is extracted by using a DNeasy Power Soil Kit (QIAGEN, Germany); The standard protocol is followed as the kit guide. The DNA from bacteria in each sample is quantified by using QIAxpert system (QIAGEN).

**Bacterial metagenomic analysis using DNA from stool samples**

Bacterial genomic DNA was amplified with 16S_V3_F (5′ - TCG TCG GCA GCG TCA GAT GTG TAT AAG AGA CAG CCT ACG GGN GGC WGC AG -3′) and 16S_V4_R (5′ - GTC TCG TGG GCT CGG AGA TGT GTA TAA GAG ACA GGA CTA CHV GGG TAT CTA ATC C -3′) primers, which are specific for V3-V4 hypervariable regions of 16S rDNA gene. The libraries were prepared using PCR products according to MiSeq System guide (Illumina, Coyoacán, Mexico City) and quantified using a QIAxpert (QIAGEN). Each amplicon is then quantified, set equimolar ratio, pooled, and sequenced on MiSeq (Illumina) according to the manufacturer’s recommendations.

**Analysis of bacterial composition in the microbiota**

Paired-end reads that matched the adapter sequences were trimmed by cutadapt version 1.1.6. The resulting FASTQ files containing paired-end reads were merged with CASPER and then quality filtered with Phred (Q) score based criteria described by Bokulich. Any reads were shorter than 350 bp and longer than 550 bp after merging, were also discarded. To identify the chimeric sequences, a reference-based chimera detection step was conducted with VSEARCH against the SILVA gold database. Next, the sequence reads were clustered into Operational Taxonomic Units (OTUs) using VSEARCH with de novo clustering algorithm under a threshold of 97% sequence similarity. The representative sequences of the OTUs were finally classified using SILVA 132 database with UCLUST (*parallel_assign_taxonomy_uclust.py* script on QIIME version *1.9.1*) under default parameters [2]. The Chao Indices, an estimator of richness of taxa per individual, were estimated to measure the diversity of each sample.

**Reference**

1 Nam SY, Kim N, Lee CS et al. Gastric mucosal protection via enhancement of MUC5AC and MUC6 by geranylgeranylacetone. Dig Dis Sci 2005;50(11):2110-2120.

2 Caporaso JG, Kuczynski J, Stombaugh J et al. QIIME allows analysis of high-throughput community sequencing data. Nat Methods 2010;7(5):335-336.
